# Consumer Engagement in Chronic Conditions Research: An Integrated Framework Informed by Recognition Theory

**DOI:** 10.1111/hex.70615

**Published:** 2026-02-22

**Authors:** Mingming Zhou, Anne Parkinson, Leanne Watts, Julie Veitch, Hanna Suominen, Jane Desborough

**Affiliations:** ^1^ National Centre for Epidemiology and Population Health (NCEPH) Australian National University (ANU) Canberra Australian Capital Territory Australia; ^2^ Consumer with lived experience of chronic condition Brisbane Queensland Australia; ^3^ Consumer with lived experience of chronic condition Canberra Australian Capital Territory Australia; ^4^ School of Computing, College of Systems & Society, ANU Canberra Australian Capital Territory Australia; ^5^ School of Medicine & Psychology, College of Science & Medicine, ANU Canberra Australian Capital Territory Australia; ^6^ Department of Computing, Faculty of Technology University of Turku Turku Finland

**Keywords:** chronic conditions, consumer engagement, consumer remuneration, framework, health research, patient and public involvement, recognition theory

## Abstract

**Background:**

Consumer engagement ensures that health research reflects lived experiences and generates outcomes relevant to those most affected. However, frameworks guiding engagement in research about chronic conditions remain limited and often lack theoretical grounding.

**Objective:**

To develop an integrated, evidence‐based framework to support consumer engagement in research about chronic conditions.

**Methods:**

We integrated findings from (1) a scoping review synthesising evidence‐based resources supporting consumer engagement in research about chronic conditions (Resource Framework) and (2) a co‐designed framework for recognising consumers' contributions to research within the Australian context (Recognition Framework). Our integration deployed the relational, structural, and symbolic domains of Honneth's recognition theory as an analytical lens and used joint displays to develop a comprehensive framework.

**Results:**

The framework demonstrates how relational, structural, and symbolic dimensions of recognition collectively support ethical and sustainable consumer engagement. Relational recognition (e.g., mutual learning, ongoing communication) strengthens interpersonal trust and shared decision‐making; structural recognition (e.g., governance policies, remuneration, reimbursement) embeds engagement within institutional systems; and symbolic recognition (e.g., authorship, formal acknowledgement) legitimises consumers' expertise within research cultures. Together, these elements provide a comprehensive foundation for supporting meaningful engagement across research practices.

**Conclusion:**

This integrated recognition theory‐informed framework offers an evidence‐based tool to inform the design and implementation of consumer engagement in research about chronic conditions. By positioning recognition for consumers' contribution as an ethical, structural, and symbolic principle, it offers a transferable framework to strengthen participatory practice and advance equity in research. While developed for chronic conditions research, the framework is likely transferable with contextual tailoring to other settings.

## 1 Introduction

Consumer engagement in research has evolved from a peripheral consultation process to a policy expectation across major health systems globally [1]. The National Institute for Health Research (NIHR) [2], Patient‐Centred Outcomes Research Institute (PCORI) [3], and Australia's National Health and Medical Research Council (NHMRC) now require evidence of meaningful consumer engagement in funded research [4]. This policy momentum reflects growing recognition that incorporating lived experience enhances the relevance, acceptability, and implementation of research outcomes [5].

Despite this policy support and commitments to partnership and shared decision‐making, meaningful engagement is not consistently achieved. Collaborations with patients and the public can unintentionally reproduce power inequities, leaving those with lived experience feeling marginalised or tokenised as ‘the most vulnerable and most disadvantaged members of the team’ [6]. This highlights a gap between aspirations for partnership and the realities of practice. Consumers may be invited into research, yet their contributions are not always valued or integrated in ways that support genuine partnership [6, 7, 8].

In this paper, we use the term ‘consumers’ to refer to individuals with lived experience of health issues, including patients, carers, and family members [9]. This terminology reflects its widespread usage in Australian research and policy. Internationally, similar roles are described as patient and public involvement (PPI) in the UK, or patient engagement in Canada and the United States. While retaining the term ‘consumer’ for consistency with our context and prior work, we aim to contribute to broader international conversations about inclusive research practices and recognition.

We adopt Harrington et al.'s (2020, p.682) definition of consumer engagement as ‘the active, meaningful, and collaborative interaction between patients and researchers across all stages of the research process, where research decision making is guided by patients' contributions as partners, recognising their specific experiences, values, and expertise.’ [10] This framing positions consumers not as passive participants or external advisors, but as co‐producers of research, whose experiential knowledge has both epistemic and ethical value.

In Australia, formal policy development of consumer engagement began with the 2002 *Statement on Consumer and Community Participation in Health and Medical Research*, followed by a 2004 model Framework and Resource Pack [4]. A 2016 revision reframed ‘participation’ as ‘involvement’, and in 2020, NHMRC released a Toolkit offering practical resources for implementation [11]. While these documents signal a strong commitment, they mainly outline high‐level principles rather than detailed, practice‐focused guidance, particularly outside clinical research contexts. Few published studies provide insights into authentic consumer engagement within the Australian research community [12].

These limitations become more significant when considering chronic conditions, a major public health challenge. Chronic or noncommunicable diseases account for 74% of global deaths, around 41 million annually, and disproportionately affect health systems in developed countries [13, 14, 15]. In Australia, six in ten adults live with at least one chronic condition, and over 90% of the non‐fatal disease burden is attributed to these conditions [16]. Such prevalence underscores the need for research that meaningfully incorporates lived experience. Evidence from Australian studies involving consumers living with multiple sclerosis and diabetes shows that consumers can act as research partners, offering unique contributions grounded in lived experience [17, 18]. Yet consumers with chronic conditions often face condition‐specific barriers, such as fluctuating symptoms and fatigue, which general frameworks do not fully address [19]. Limited resources exist in Australia to support tailored, condition‐specific engagement [12, 17, 20].

To respond to these gaps, we conducted a multi‐phase mixed methods study to develop evidence‐based resources for engaging consumers in research about chronic conditions. The study included a scoping review, a cross‐sectional survey, and six deliberative workshops, reported in detail elsewhere [9, 21, 22]. Findings from the scoping review informed the development of a resource framework to support consumer engagement in research about chronic conditions (hereafter referred to as the Resource Framework, Supplementary Figure 1), which included six interrelated domains: (1) reciprocal learning, (2) fostering a supportive environment, (3) providing training to build capacity, (4) acknowledging consumers' contributions, (5) using resources to facilitate engagement, and (6) evaluating engagement impact. Among these, ‘acknowledging consumer contributions' emerged as a critical yet underexplored area. Building on this work, we subsequently co‐designed a recognition framework, using evidence from a cross‐sectional survey [22] and deliberative workshops, to guide how consumers' contributions are acknowledged in Australian health and medical research, including both financial and non‐financial methods (hereafter referred to as the Recognition Framework, Supplementary Figure 2) [21]. This initiative represented an important step towards standardising recognition practices, providing guidance on decision‐making, remuneration rates, and financial and non‐financial recognition approaches.

The Resource Framework synthesises empirical evidence for supporting consumer engagement in chronic conditions research, but it lacks an explicit theoretical foundation. Conversely, the Recognition Framework provides specific guidance on valuing contributions but does not offer the practical structures required to embed meaningful engagement in everyday research practice. Together, these limitations reveal a research gap: meaningful engagement cannot be achieved through empirical evidence alone without a theoretical foundation, nor through procedural guidance without practical mechanisms for implementation. A conceptual framework that aligns empirical practice with ethical principles is required to guide researchers and consumers in their approach to consumer engagement in research about chronic conditions.

To address this gap, we draw on recognition theory as a lens for understanding how consumers' contributions can be meaningfully valued and sustained in research about chronic conditions. Meaningful consumer engagement meets fundamental human needs for recognition, respect, and value [23]. In research, consumers' contributions encompass both experiential knowledge and relational labour, which require acknowledgement to support genuine participation. Recognition theory, particularly Honneth's typology of love, respect, and social esteem, offers a robust philosophical basis for understanding consumer engagement as an ethical relationship grounded in equity, dignity, and social justice [24].

Building on findings from our multi‐phase mixed methods study, this paper develops an integrated theory‐informed framework that synthesises the Resource and Recognition Frameworks into a coherent structure. The integrated framework offers an overarching approach for researchers, organisations, and policymakers seeking to operationalise recognition as the foundation of ethical and meaningful consumer engagement in research about chronic conditions.

## 2 Methods

### 2.1 Design

This study used a multi‐phase, mixed methods design, guided by a participatory worldview [25]. This perspective views reality as socially constructed and politically situated, emerging through collaborative action, and supports the iterative integration of stakeholder input across research phases [26, 27]. The study aimed to answer the overarching research question: how can consumer engagement in research about chronic conditions be better supported? Each phase generated complementary evidence, which was synthesised to develop an integrated framework capturing how consumers' contributions can be supported and recognised across research contexts.

**FIGURE 1:**
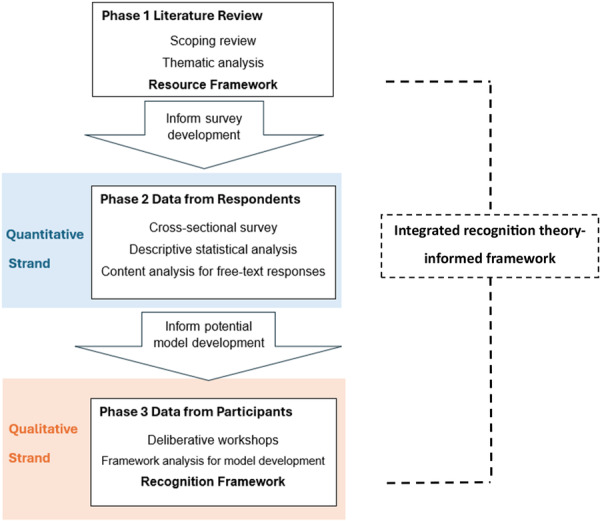
The multi‐phase, mixed methods design of this study.

As shown in Figure 1, the study was conducted in three phases. Phase 1 involved a scoping review of resources to support consumer engagement in research about chronic conditions [9]. Evidence was synthesised to inform the development of an overarching Resource Framework. Phase 2 involved the co‐design of a survey that was distributed to research organisations across Australia to gain insight in perspectives and current practices of recognising consumers' contributions to research [22]. Phase 3 incorporated evidence from phases 1 and 2 for consideration by consumers and researchers in a series of deliberative workshops. These findings informed the development of the Recognition Framework, to support financial and non‐financial forms of recognition [21].

### 2.2 Theoretical Framework

Honneth's theory of recognition provides a robust lens for understanding how consumer engagement can be operationalised in ways that attend to interpersonal relationships, institutional arrangements, and social valuation [24, 28, 29]. His three categories: love (interpersonal connection), rights (legal recognition), and social esteem (solidarity), were applied in this study as relational, structural, and symbolic dimensions of recognition. Drawing on applications of recognition theory in other social research fields [28, 29], Table 1 summarises how these dimensions were mapped to operational considerations for consumer engagement in research.

**TABLE 1:** Mapping Honneth's recognition theory to the operational dimensions of consumer engagement in research.

Recognition theory		Operational dimension
Love/Care	Private sphere	Relational recognition:
		Captures the interpersonal and emotional aspects of recognition, such as trust‐based collaboration, ongoing communication, and mutual learning between researchers and consumers.
Rights	Legal sphere	Structural recognition:
		Captures formal, procedural, and rights‐based mechanisms, including governance policies, overarching frameworks, and formal decision‐making processes.
Social esteem	Solidarity sphere	Symbolic recognition:
		Captures social and symbolic aspects embedded in norms and values, such as authorship, acknowledgement in outputs, and institutional recognition of experiential knowledge.

### 2.3 Integration Approach

In this study, findings from two components: the Resource Framework derived from the scoping review (Phase 1) and the Recognition Framework generated from the cross‐sectional survey and deliberative workshops (Phases 2–3), were systematically integrated. Integration was guided by recognition theory, which provided a conceptual lens for understanding how relational, structural, and symbolic forms of recognition interact to support meaningful consumer engagement.

We used joint displays, a mixed methods integration technique widely applied in research, to visually and analytically align the resource themes identified in the scoping review with recognition categories from the survey and workshops [30]. This approach facilitated the identification of conceptual overlaps, complementarities, and interactions between the two datasets, enabling a coherent synthesis. The joint displays also provided a structured platform for iterative discussion and interpretation, ultimately informing the development of an integrated, theory‐based framework that illustrates how consumers' contributions can be valued and operationalised across research contexts.

## 3 Results

**FIGURE 2:**
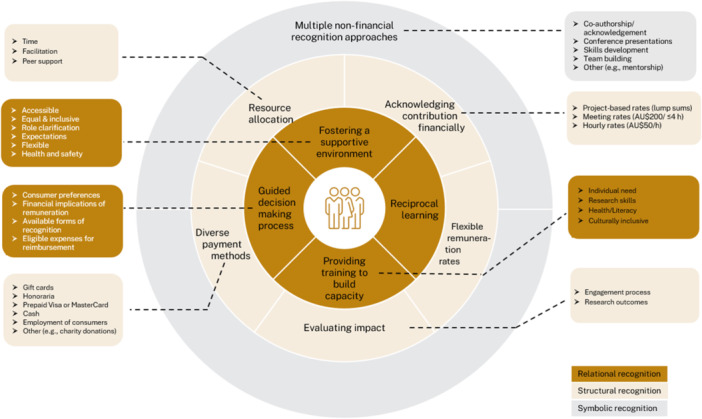
Integrated recognition theory‐informed framework to support consumer engagement in research about chronic conditions.

Integration of the Resource Framework and Recognition Framework through joint displays (Table 2) produced an integrated framework informed by recognition theory (Figure 2 *The recognition theory‐informed framework to support consumer engagement in research about chronic conditions*). The integration showed that the Resource Framework mapped across all three recognition domains (relational, structural, and symbolic), whereas the Recognition Framework provided more detailed, operational guidance focused on the structural and symbolic domains, particularly in relation to financial and non‐financial recognition of consumer contributions.

**TABLE 2:** Joint display of the resource framework and recognition framework using three dimensions of Honneth's recognition theory.

Recognition theory	Resource Framework	Recognition Framework	Interpretation using recognition theory
**Love/Care** **Interpersonal connection** **Relational dimension**	**Reciprocal learning** Consumers: personal growth and improved management skills. Researchers: deep understanding of the end users' needs. **Fostering a supportive environment** a. Optimising accessibility b. Equal and inclusive atmosphere c. Open discussion about roles d. Setting realistic expectations e. Flexible engagement methods f. Ensure health and safety **Providing training to build capacity** a. Identify individual needs	**Guided decision‐making process** a. Which forms of recognition do engaged consumers prefer? b. What potential financial implications should consumers consider when opting for financial recognition?	Reciprocal learning, a supportive environment, and tailored capacity building exemplify relational recognition by fostering trust, mutual learning, and meaningful interpersonal engagement. These elements promote accessibility, inclusivity, dialogue, and flexible support, ensuring consumers' contributions are valued not only for their outputs but also for the relational connections they bring to research.
**Rights** **Legal recognition** **Structural dimension**	**Providing training to build capacity** b. Research skills c. Health/Literacy improvement d. Cultural inclusiveness **Using resources to facilitate engagement** a. Time investment b. Coordinator or facilitator c. Peer support. **Evaluating engagement impact** a. Impact on the engagement process b. Impact on research outcomes **Acknowledging consumers' contributions** a. Financial remuneration Financial recognition includes payments for advisory roles, meeting expenses, translation services, multimedia tools, and transportation, childcare and refreshments costs.	**Guided decision‐making process** c. What forms of recognition will be offered to consumers participating in this project? d. What expenses are eligible for reimbursement under this project? **Flexible remuneration rates** a. Project‐based rates (lump sums based on project scope) b. Meeting rates (AU $200/meeting ≤ 4 h) c. Hourly rates (AU $50/h). **Diverse payment methods** a. Gift cards b. Bank transfers (honoraria) c. Prepaid Visa or MasterCard d. Cash e. Employment of consumers f. Other options, such as donations to a designated charity	Capacity building, resource allocation, evaluation of impact, and financial recognition exemplify structural recognition by embedding formal, procedural, and rights‐based mechanisms into research practice. These elements ensure that consumers and researchers are supported through structured training, dedicated resources, systematic feedback, and transparent remuneration processes. Together, they operationalise ethical, consistent, and equitable consumer engagement within organisational policies and governance frameworks.
**Social esteem** **Solidarity** **Symbolic dimension**	**Acknowledging consumers' contribution** b. Non‐financial recognition methods, such as co‐authorship Non‐financial recognition includes appropriately acknowledging the contributions of consumer partners, such as offering opportunities for co‐authorship.	**Multiple non‐financial recognition approaches** a. Co‐authorship or acknowledgement on academic outputs b. Co‐/presentations at conferences c. Skills development opportunities d. Team building e. Other options (e.g., public recognition or mentorship)	Non‐financial recognition, including co‐authorship, conference co‐/presentations, skills development, and team‐building opportunities, exemplifies symbolic recognition by publicly acknowledging consumers' expertise and lived experience. These approaches embed recognition within social and institutional norms, legitimising contributions and fostering a respectful and ethical research culture. Together, they ensure consumers' contributions are acknowledged in meaningful and socially visible ways.

### 3.1 Recognition Theory Dimension 1: Relational Recognition of Consumer Engagement

Relational recognition (love/care) captures the interpersonal and emotional aspects of recognition reflected in trust‐based collaboration, ongoing communication, and mutual learning between researchers and consumers. In the joint display (Table 2), three elements of the Resource Framework are mapped primarily to this domain: reciprocal learning, fostering a supportive environment, and capacity building tailored to individual needs. Reciprocal learning was represented as mutual benefit, with consumers reporting personal growth and improved management skills, and researchers gaining a deeper understanding of end users' needs. A supportive environment comprised strategies to optimise accessibility, promote an equal and inclusive atmosphere, support open discussion about consumer roles, set realistic expectations, use flexible engagement methods, and attend to consumers' health and safety. Capacity building in this domain emphasised identifying individual needs through mentorship, dialogue, and co‐learning as the basis for tailored support.

The Recognition Framework contributed to this domain through the guided decision‐making process. In Table 2, this process included a structured discussion of which forms of recognition consumers prefer, and consideration of potential implications when consumers opt for financial recognition. These elements reflect relational processes that support ongoing communication and shared decision‐making when determining recognition approaches within a project.

### 3.2 Recognition Theory Dimension 2: Structural Recognition of Consumer Engagement

Structural recognition (rights‐based recognition) captures formal and procedural mechanisms that embed consumer engagement within governance arrangements, overarching frameworks, and decision‐making processes. In the joint display (Table 2), four elements of the Resource Framework are mapped primarily to this domain: capacity building, resource allocation, evaluating impact, and financial recognition. Capacity building was represented through structured training programs targeting research skills, health literacy, and cultural inclusiveness. Resource allocation included dedicated time for researchers, providing peer support for consumers, and employing engagement facilitators. Evaluation was captured through formal assessment of engagement impact on the engagement process and research, including outcomes for consumers and researchers. Financial recognition was represented as remuneration and reimbursement to support engagement, including payments for remuneration and coverage of practical costs such as transport, childcare, refreshments, and other participation‐related expenses.

The Recognition Framework contributed more detailed operational guidance on implementing structural recognition. In Table 2, this included a guided decision‐making process to determine which forms of recognition are available and which expenses are eligible for reimbursement within a given project. It also specified flexible remuneration rate options (project‐based lump sums, meeting‐based rates, and hourly rates) and diverse payment methods (e.g., gift cards, bank transfers/honoraria, prepaid cards, cash, employment arrangements, or alternative options such as donations to a designated charity). Together, these elements describe the procedural and resourcing infrastructure through which consumer engagement can be implemented in a consistent and transparent way.

### 3.3 Recognition Theory Dimension 3: Symbolic Recognition of Consumer Engagement

Symbolic recognition (social esteem/solidarity) captures the social and symbolic aspects of consumer engagement that are embedded in research norms and values, including authorship, acknowledgement in outputs, and the institutional valuing of experiential knowledge. In the joint display (Table 2), the Resource Framework contributed a core element mapped to this domain: acknowledging consumers' contributions through non‐financial recognition, such as appropriately acknowledging consumer partners or offering co‐authorship where relevant.

The Recognition Framework provided a broader set of non‐financial recognition options within this domain. These included co‐authorship or acknowledgement on academic outputs, co‐/presentations at conferences, skills development opportunities, team building activities, and other forms of recognition (e.g., public recognition or mentorship). Together, these elements describe the range of practices through which consumer contributions may be made visible and valued within research outputs and research team culture.

## 4 Discussion

### 4.1 Summary of Key Findings

This study integrated two empirically developed frameworks using recognition theory as an analytical lens: a Resource Framework identifying evidence‐based supports for consumer engagement in research about chronic conditions [9], and a Recognition Framework specifying how contributions may be acknowledged within the Australian context [21]. The integration generated an integrated framework that organises engagement supports and recognition practices across three recognition domains (relational, structural, and symbolic). The joint‐display mapping identified that the Resource Framework contributed across all three domains, whereas the Recognition Framework added more detailed operational guidance in the structural and symbolic domains.

### 4.2 Interpretation Using Recognition Theory

Based on the joint displays in Table 2, integrating the Resource and Recognition Frameworks (see Results section), Honneth's theory of recognition provides an interpretive lens for understanding how different forms of recognition may shape the quality and sustainability of consumer engagement. The relational elements identified in the joint display: reciprocal learning, supportive environments, and capacity building tailored to individual needs, function as conditions for trust, role clarity, and ongoing communication. When these relational conditions are weak, engagement may be limited to procedural involvement rather than experienced as a partnership, even where structural arrangements are in place. Guided decision‐making further reinforces relational recognition by positioning consumers as equal partners in shaping how recognition is implemented in practice.

Structural elements: formal training, dedicated resourcing (e.g., coordinator roles, time investment, and peer support), evaluation processes and financial recognition, operate as rights‐ and procedure‐based supports that reduce reliance on individual goodwill and enable more consistent implementation across projects. Where structural recognition is absent or ad hoc (e.g., unclear governance, inconsistent resourcing, or reimbursement processes that are not transparent), engagement is more likely to be inequitable, difficult to sustain, and less clear in expectations and accountability.

Symbolic recognition elements, such as co‐authorship or acknowledgement on outputs, co‐presentations, skills development, team‐building opportunities, and public recognition or mentorship, shape the visibility and perceived legitimacy of consumers' experiential knowledge within research cultures. When symbolic recognition is limited, consumers may contribute but have fewer opportunities for their expertise to be treated as credible and visible within academic norms.

Taken together, recognition theory highlights how deficits in any one domain can create predictable vulnerabilities in engagement, and why strengthening recognition across all three domains (relational, structural and symbolic) is likely to support more robust partnership practices [6].

Furthermore, the integration yielded three overarching insights. First, consumer engagement requires simultaneous attention to the relational, structural, and symbolic dimensions of recognition, and efforts that focus on only one dimension may leave predictable gaps in practice. Second, these dimensions are complementary and operate at different levels: interpersonal, organisational and cultural, providing a structured way to identify where engagement support is most constrained in a given context. Each dimension addresses a distinct aspect of recognition, and all three are necessary for meaningful engagement. Third, different stakeholders, including researchers, organisations, and policymakers, have distinct but complementary roles in supporting consumer engagement across these dimensions.

### 4.3 Addressing the Research Gap

*The recognition theory‐informed framework to support consumer engagement in research about chronic conditions* responds to the following gaps identified in current practice. First, it bridges the aspiration‐reality gap in partnership practices by operationalising abstract commitments to ‘partnership’ through concrete mechanisms across relational, structural, and symbolic dimensions. Researchers can now ask not simply ‘are we partnering?’ but ‘are we building partnership through reciprocal learning, fair remuneration, and meaningful non‐financial acknowledgement approaches?’ This specificity transforms vague policy aspirations into actionable practices.

Second, the framework provides the practical, context‐specific guidance that Australian policy documents currently lack. While the NHMRC's Statement and Toolkit outline high‐level principles, they offer limited direction for operationalisation, particularly outside clinical trial contexts [4, 11]. Our framework provides more operational guidance by providing actionable strategies tailored to chronic condition research contexts, with explicit attention to condition‐specific barriers such as fluctuating symptoms, fatigue, and the need for flexible engagement consideration [19]. For instance, the relational dimension's emphasis on health and safety considerations directly responds to the reality that consumers with chronic conditions may experience unpredictable symptoms that require flexible, appropriate accommodations.

Third, the framework addresses the absence of condition‐specific resources in Australia. While international frameworks, such as the patient engagement framework from SPOR in Canada and INVOLVE commonly used in the UK [31, 32] have advanced the field, Australia has lacked a comparable evidence‐based framework to support consumer engagement tailored to the local context and research about chronic conditions. Our framework, developed through mixed methods research with Australian consumers and researchers, provides this missing resource while contributing to international conversations about inclusive consumer engagement practices.

Fourth, and most importantly, the framework provides a stronger conceptual rationale that neither the Resource Framework nor the Recognition Framework alone could offer. The Resource Framework identified what supports are needed, but not why they matter or how they relate to ethical principles. The Recognition Framework specified how to acknowledge contributions but lacked the broader conceptual architecture connecting recognition to engagement quality. By using recognition theory as an analytical lens to integrate both frameworks, the integrated framework reveals the mechanisms through which engagement practices either support or undermine dignity, equity, and justice. This theoretical grounding enables stakeholders to understand not only what to do but also why certain practices matter and how to adapt these principles to specific contexts.

### 4.4 Implementation in Practice

Implementation of *the recognition theory‐informed framework to support consumer engagement in research about chronic conditions* may require a phased, stakeholder‐coordinated approach, with distinct but complementary roles for researchers, organisations, and policymakers.

For researchers, the framework's relational dimension offers an accessible entry point, requiring minimal resources beyond time and genuine commitment. Our findings suggest that relational recognition is foundational; without it, structural and symbolic mechanisms may be experienced as less meaningful. This aligns with review evidence highlighting that trusting relationships are essential for consumer partners to be regarded as equal members of research teams [33]. Specific strategies may include: clarifying consumers' roles at project outset; establishing regular check‐ins to discuss recognition preferences and expectations; building flexibility into timelines to accommodate health fluctuations; and creating supportive environments through accessible communication and inclusive interactions [9]. Early and transparent discussions about recognition, including forms of acknowledgement and remuneration options, strengthen trust and reinforce consumers' sense of value. However, researchers cannot enact meaningful recognition alone; relational efforts without structural support risk placing unsustainable emotional labour on individuals and perpetuating the power imbalances that meaningful engagement aims to address.

Organisations, therefore, need to embed structural recognition through formal policies and resource allocation. Our findings align with existing literature demonstrating that inadequate resources, including funding, staffing, and institutional guidance, represent persistent barriers to meaningful engagement [34, 35, 36, 37]. Practical implementation steps include: developing institutional remuneration guidelines that specify remuneration rates (informed by *the recognition theory‐informed framework to support consumer engagement in research about chronic conditions*), eligible expenses, and payment options; allocating dedicated full‐time equivalent positions for engagement coordination rather than adding engagement responsibilities to already burdened research staff; and establishing evaluation approaches that assess engagement impact across multiple domains, including impact on research processes, outcomes, researchers, consumers, and broader communities [7, 38, 39]. Organisations should anticipate that meaningful engagement requires investment in funding, dedicated staff time, appropriate budgets for remuneration and reimbursement, training resources, and evaluation systems. Without this structural foundation, even well‐intentioned relational efforts by individual researchers cannot achieve sustainable and equitable engagement.

For policymakers, the framework highlights the need for systemic support that enables, rather than merely expects, engagement. Research funders can incentivise multi‐dimensional recognition by: requiring applicants to detail how they will operationalise each dimension of this framework to support engagement; providing dedicated engagement budgets separate from research activity costs; assessing proposals on the quality of engagement plans using criteria derived from this framework; and supporting evaluation research on engagement impact. At a systemic level, embedding recognition principles into national frameworks, such as NHMRC and ARC guidelines, would show that meaningful engagement is treated as integral to research excellence. Policymakers may consider how funding structures can better support the time and resources needed for authentic partnership, moving beyond symbolic inclusion to genuine co‐production.

### 4.5 Strengths and Limitations

Several strengths enhance this framework's potential contribution to research practice and policy. First, it is empirically grounded in multi‐phase mixed‐method research, ensuring robust evidence‐based recommendations. Second, the framework was co‐designed with consumers and researcher organisations rather than imposed top‐down, enhancing its practical relevance and applicability. Third, recognition theory provides conceptual rigour, connecting pragmatic strategies to the fundamental ethical principles of dignity, equity, and justice. Finally, it fills a critical gap in resources supporting consumer engagement in research about chronic conditions.

However, several limitations warrant consideration. First, while the framework was developed through rigorous methods, it has not yet been tested in practice. Empirical validation is needed to assess whether implementing the framework improves engagement experiences and research outcomes. Second, the framework's development in the Australian context, which was informed by evidence and stakeholder input, focused on chronic conditions research. While the relational, structural, and symbolic domains are likely applicable across many areas of health research, transferability to other settings may require context‐specific adaptation to different health systems, funding structures, cultural norms around consumer roles, and terminology (e.g., consumer vs. patient, and consumer engagement vs. patient and public involvement). Third, while the framework emphasises evaluation as a component of structural recognition, it does not provide specific metrics for assessing engagement quality or distinguishing meaningful from performative practices. Future research should apply or develop validated instruments for evaluation. Despite these limitations, the framework provides a theoretically informed and practically oriented foundation for strengthening consumer engagement in research, with chronic conditions serving as a salient development context and test case.

## 5 Conclusion

Consumer engagement in research has evolved from a peripheral aspiration to a policy expectation, yet meaningful engagement remains inconsistent in practice. *The recognition theory‐informed framework to support consumer engagement in research about chronic conditions* addresses critical gaps in supporting consumer engagement in research about chronic conditions. The lens of Honneth's recognition theory demonstrates that meaningful engagement requires attention to three complementary dimensions: relational recognition, enacted through interpersonal relationship‐building and mutual respect; structural recognition, embedded through policies, resources, and governance mechanisms; and symbolic recognition, reflected and shaped by the broader research culture and publicly legitimising consumers' lived experiences and contributions. This multi‐dimensional approach moves beyond procedural checklists to addressing the fundamental ethical principles of dignity, equity and justice that underpin genuine partnership.

Although developed in the context of chronic conditions research, the framework is likely relevant beyond this setting. Chronic conditions research often amplifies requirements for sustained and flexible engagement, given longer time horizons, ongoing interactions with health services and research systems, and fluctuating symptoms or capacity, making it a useful context in which to develop and interpret recognition requirements. At the same time, the framework's domains reflect cross‐cutting conditions for consumer engagement and may be adapted to other research contexts with appropriate tailoring.

Further work is needed to understand how the framework functions in real‐world settings. Prospective implementation studies could test its impact on engagement processes, research quality, and partnership sustainability. Developing practical tools, such as implementation checklists, policy templates, and training resources, would support its adoption, ideally through co‐design with researchers and consumers. Future studies should also examine how the framework can be adapted to advance equity, particularly for indigenous people, culturally and linguistically diverse communities, and other groups historically marginalised in research, aligning with recognition theory's concern with justice.

More broadly, this framework contributes to international efforts to shift research cultures by framing consumer engagement not as a methodological requirement, but as an ethical imperative. By integrating relational, structural, and symbolic dimensions of recognition, it provides a transferable framework to guide more inclusive, relevant, and responsive research for those most affected, the consumer.

## Author Contributions

**Mingming Zhou:** conceptualisation, methodology, data analysis, writing – original draft, review and editing. **Anne Parkinson:** supervision, methodology, validation, writing – review and editing. **Leanne Watts:** lived experience expert, validation, writing – review and editing. **Julie Veitch:** lived experience expert, validation, writing – review and editing. **Hanna Suominen:** methodology, writing – review and editing. **Jane Desborough:** supervision, methodology, data analysis, validation, writing – review and editing.

## Funding

The authors received no specific funding for this work.

## Patient or Public Contribution

Consumers with lived experience of chronic conditions were involved in the refinement and interpretation of the integrated framework. Two consumer partners contributed substantive feedback on the final framework and are included as co‐authors in recognition of their intellectual contributions.

## Conflicts of Interest

The authors declare no conflicts of interest.

## Supporting information

Supporting Figure 1 Resource Framework.

Supporting Figure 2 Recognition Framework.

## Data Availability

Data sharing is not applicable to this manuscript as no datasets were generated or analysed from this study.
