# Supplementary material for: Consumer Engagement in Chronic Conditions Research: An Integrated Framework Informed by Recognition Theory
Source: Health Expect. 2026 Feb 22;29(1):e70615. doi: 10.1111/hex.70615 (PMC12928018; doi:10.1111/hex.70615)

Resource framework to support consumer engagement in research about people living with chronic conditions

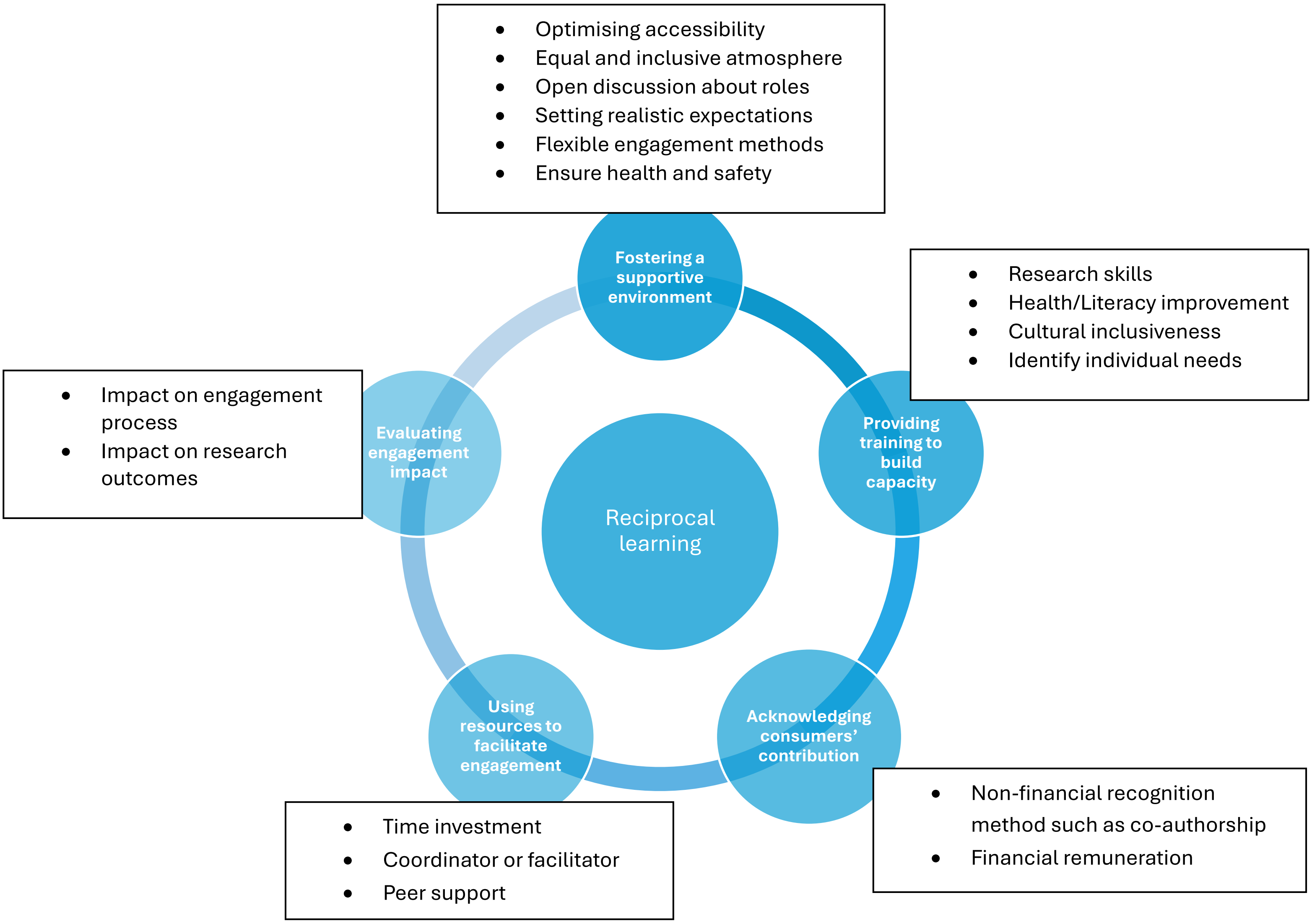

Supplement: Supplementary file 1 — Supporting Figure 1 Resource Framework. [file HEX-29-e70615-s002.pdf]
