# Supplementary material for: Consumer Engagement in Chronic Conditions Research: An Integrated Framework Informed by Recognition Theory
Source: Health Expect. 2026 Feb 22;29(1):e70615. doi: 10.1111/hex.70615 (PMC12928018; doi:10.1111/hex.70615)
Supplement: Supplementary file 2 — Supporting Figure 2 Recognition Framework. [file HEX-29-e70615-s001.pdf]

Recognition Framework to support financial and non-financial recognition of consumers' contribution to health research

Decision-making considerations →

What forms of recognition will be offered to consumers participating in this project?

What expenses are eligible for reimbursement under this project?

Which forms of recognition do engaged consumers prefer?

What potential financial implications should consumers consider when opting for financial recognition?

Financial recognition methods

Non-financial recognition approaches →

- Co-authorship or acknowledgement on academic outputs
- Co-presentations at conferences
- Opportunities for skills development
- Team building
- Other (such as public recognition and consumer mentorship)

Payment Rates

Payment methods →

- Gift card
- Bank transfer (Honoraria)
- Pre-paid Visa or Master card
- Cash
- Employment of consumers
- Other (such as donation to designated charity)

Project Rate

Lump sums based on project scope

Meeting Rate

$200 per meeting (⩽4 hours)

Hourly Rate

$50/hour
